# An Opportunistic Survey Reveals an Unexpected Coronavirus Diversity Hotspot in North America

**DOI:** 10.3390/v13102016

**Published:** 2021-10-07

**Authors:** Hon S. Ip, Kathryn M. Griffin, Jeffrey D. Messer, Megan E. Winzeler, Susan A. Shriner, Mary Lea Killian, Mia K. Torchetti, Thomas J. DeLiberto, Brian R. Amman, Caitlin M. Cossaboom, R. Reid Harvey, Natalie M. Wendling, Hannah Rettler, Dean Taylor, Jonathan S. Towner, Casey Barton Behravesh, David S. Blehert

**Affiliations:** 1 United States Geological Survey, National Wildlife Health Center, Laboratory Services Branch. Madison, WI 53711, USA; kgriffin@usgs.gov (K.M.G.); jdmesser@usgs.gov (J.D.M.); mwinzeler@usgs.gov (M.E.W.); dblehert@usgs.gov (D.S.B.); 2 Wildlife Services, National Wildlife Research Center, United States Department of Agriculture, Fort Collins, CO 80521, USA; susan.a.shriner@usda.gov (S.A.S.); thomas.j.deliberto@usda.gov (T.J.D.); 3 National Veterinary Services Laboratories, Diagnostic Virology Laboratory, United States Department of Agriculture, Ames, IA 50010, USA; mary.l.killian@usda.gov (M.L.K.); mia.kim.torchetti@usda.gov (M.K.T.); 4 Centers for Disease Control and Prevention, National Center for Emerging and Zoonotic Infectious Diseases, Atlanta, GA 30329, USA; cxx1@cdc.gov (B.R.A.); nrm9@cdc.gov (C.M.C.); iez1@cdc.gov (R.R.H.); pme8@cdc.gov (N.M.W.); jit8@cdc.gov (J.S.T.); dlx9@cdc.gov (C.B.B.); 5 Utah Department of Health, Salt Lake City, UT 84114, USA; hrettler@utah.gov; 6 Utah Department of Agriculture and Food, Salt Lake City, UT 84116, USA; djtaylor@utah.gov

**Keywords:** coronavirus, SARS-CoV-2, mink, mink farm, zoonosis, spill-over, pandemic

## Abstract

In summer 2020, Severe Acute Respiratory Syndrome Coronavirus 2 (SARS-CoV-2) was detected on mink farms in Utah. An interagency One Health response was initiated to assess the extent of the outbreak and included sampling animals from on or near affected mink farms and testing them for SARS-CoV-2 and non-SARS coronaviruses. Among the 365 animals sampled, including domestic cats, mink, rodents, raccoons, and skunks, 261 (72%) of the animals harbored at least one coronavirus. Among the samples that could be further characterized, 127 alphacoronaviruses and 88 betacoronaviruses (including 74 detections of SARS-CoV-2 in mink) were identified. Moreover, at least 10% (*n* = 27) of the coronavirus-positive animals were found to be co-infected with more than one coronavirus. Our findings indicate an unexpectedly high prevalence of coronavirus among the domestic and wild free-roaming animals tested on mink farms. These results raise the possibility that mink farms could be potential hot spots for future trans-species viral spillover and the emergence of new pandemic coronaviruses.

## 1. Introduction

Coronaviruses are a group of viruses with a diverse host range within the family *Coronaviridae*, subfamily *Orthocoronavirinae*. Four genera are recognized within this subfamily, *Alphacoronavirus*, *Betacoronavirus*, *Gammacoronavirus*, and *Deltacoronavirus* [1]. In late 2019, a novel coronavirus (Severe Acute Respiratory Syndrome Coronavirus-2, SARS-CoV-2) emerged in China and became a pandemic virus [2]. This marks the third time in less than two decades that a coronavirus of animal-origin has acquired the ability to infect people. SARS-CoV-2 was preceded by the original Severe Acute Respiratory Syndrome Coronavirus (SARS-CoV; emergence in 2003) [3] and the Middle Eastern Respiratory Syndrome Coronavirus (MERS-CoV; emergence in 2012) [4]. All three viruses are postulated to have an ultimate origin in coronaviruses from bats, the first two of which underwent a period of adaptation in an intermediate host that improved their ability to transmit among non-bat hosts [5]. For SARS-CoV, the intermediate host was the masked palm civet (*Paguma larvata*) and possibly other “wild” animals that may be found in Asian wet markets; and for MERS-CoV the intermediate hosts are camels and other camelids [5]. In contrast, no intermediate host has yet been conclusively identified for SARS-CoV-2, and the continued and wide-spread transmission of SARS-CoV-2 among people following the closing of the South China Seafood Wholesale Market in Wuhan on Jan 1, 2020, may indicate that the virus had already adapted to efficiently transmit between people prior to its identification on Jan 7, 2020 [2].

Coronaviruses have been identified in many species of mammals. Some, such as Porcine Epidemic Diarrhea Virus (PEDV) and Porcine Respiratory Coronavirus (PRCV) are economically important pathogens [6]. The introduction of PEDV into the U.S. in 2014 is estimated to have cost a between $900 million and $1.8 billion [7]. Mice, voles, and rats harbor coronaviruses that cause mainly gastrointestinal infections, but the murine coronavirus, originally called Mouse Hepatitis Virus (MHV), provides indication of tissue tropism abilities outside of the intestinal tract. Additionally, domestic ferrets (*Mustela putorius*; family *Mustelidae*) have long been used in the laboratory as an animal model for human respiratory viruses, including coronaviruses such as SARS-CoV-2 [8]. American mink (*Neovison vison*) also belongs to the family *Mustelidae* and were thus hypothesized to be susceptible to SARS-CoV-2. Subsequently, SARS-CoV-2 infection of American mink was first detected in mink farms in the Netherlands [9]. To date, SARS-CoV-2 infection of farmed American mink has been documented in a total of 12 countries, indicating that this species, especially under captive conditions, is highly susceptible to infection by this novel pandemic virus [9,10].

In the present study, we analyzed samples from animals trapped as part of a collaborative One Health investigation involving the Utah Department of Agriculture and Food (UDAF), Utah Department of Health (UDH), Utah Division of Wildlife Resources, Centers for Disease Control and Prevention (CDC), and the U.S. Department of Agriculture (USDA) to understand the scope of SARS-CoV-2 outbreaks on nine mink farms in three Utah counties. Various tissues were examined for presence of SARS-CoV-2 and other coronaviruses. Together, we identified hundreds of animal coronaviruses from six species sampled, revealing unexpected coronavirus diversity and prevalence surrounding infected mink farms. These data contribute to an improved understanding of the coronavirus diversity in wild mammals of the United States and begin to inform risk for coronavirus spillover and potential recombination in wildlife and other free-ranging animals surrounding infected mink farms. Additional epidemiologic and laboratory testing data regarding the human and animal SARS-CoV-2 investigations on the Utah mink farms are planned.

## 2. Materials and Methods

Trapping, animal handling, euthanasia, and tissue collection were conducted by CDC and USDA personnel in accordance with Institutional Animal Care and Use Protocol 3104BARMULX, and as described by Shriner et al. [10]. Farmed mink (*Neovison vison*) were sampled on nine farms by collecting mink that were found dead each morning during the field investigation. Additionally, mink on two farms were randomly selected for euthanasia and samples were collected during necropsy. Rodents and meso-predators were captured using Sherman and Tomahawk traps, respectively, within a 3- to 5-km radius surrounding each affected farm. Two moribund peri-domestic feral cats (*Felis catus*) were euthanized and samples were collected during necropsy. A total of 365 mammals, including 251 farmed mink, 98 rodents (47 deer mice, *Peromyscus maniculatus*, and 51 house mice, *Mus musculus*), and 16 meso-carnivores (cats, mink, raccoons (*Procyon lotor*), and skunks (*Mephitis mephitis*) were collected. Following euthanasia, lung, liver, spleen, heart, kidney, small intestine, colon, and rectum were collected from each carcass, frozen, and shipped to the U.S. Geological Survey National Wildlife Health Center (NWHC) on dry ice. In the laboratory, a 10% (weight:volume) homogenate of each tissue was prepared by adding a suitable volume of viral transport medium [11], and homogenizing by bead beating with an MP FastPrep (ThermoFisher, Waltham, USA) or a Tekmar Seward stomacher (Seward Laboratory Systems, Bohemia, USA). The homogenates were clarified by centrifugation at 1000x g for 30 min, and viral RNA was recovered by extraction of 50 µL of the supernatant with the Ambion MagMax Viral 96 RNA extraction kit using a KingFisher robotic platform according to manufacturer’s instructions (ThermoFisher, Waltham, USA).

### 2.1. SARS-CoV-2 Detection

The presence of the SARS-CoV-2 viral RNA was detected using the CDC real-time reverse transcription PCR targeting the nucleocapsid (N1) gene with 5 µL of the extracted RNA in a 20 µL reaction with AgPath-ID One Step master mix (Life Technologies, Carlsbad, USA) [12].

### 2.2. Pan-Coronavirus Testing

First strand cDNA was synthesized from 5 µL of the extracted RNA by the Maxima H Minus RT kit (ThermoFisher, Waltham, USA) using random hexamers in a 20 µL reaction according to manufacturer instructions. A 25 µL broad-specificity coronavirus PCR targeting the RNA-dependent RNA polymerase (RdRp) gene using 5 µL of the first strand cDNA reaction was performed according to Hu et al. [13], except that PowerTrack Master Mix (ThermoFisher, Waltham, USA) was used as the DNA polymerase. 2.5 µL of the PCR reaction was reamplified in a 25 µL nested PCR reaction with GoTaq (Promega, Madison, USA) using primers and cycling conditions according to Falcon et al. [14]. Amplification products from the final PCR reaction were examined on a 1% agarose gel, and samples exhibiting the expected 512 basepair size were submitted for Sanger sequencing at Functional Biosciences (Madison, USA). 

### 2.3. Phylogenetic Analysis

The resulting sequences were manually edited in Sequencher (GeneCode, Ann Arbor, USA), primer sequences removed and aligned using Clustal W in MEGA X. The maximum likelihood phylogenetic tree was generated using IQ-Tree (https://www.hiv.lanl.gov/content/sequence/IQTREE/iqtree.html). ModelFinferPlus was used to identify the best substitution model. Branch support was analyzed by three different methods: SH-aLRT, aBayes and UFBoot with 1000 bootstraps. The resultant tree was visualized with iTol (https://itol.embl.de/) and annotated with the host species of origin. Only the SH-aLRT branch support statistic is displayed for the sake of clarity but all three methods had similar statistical support. Taxonomic relationships to major coronavirus genera [1], were assigned using reference sequences from GenBank and trimmed to correspond to the amplicon region.

## 3. Results

### 3.1. Overall Findings

Multiple internal organs were tested for presence of coronavirus from 365 animals, including 98 rodents and 267 meso-carnivores (252 American mink, 6 raccoons, 7 striped skunks, and 2 feral domestic cats; Table 1 and Supplementary Table S1).

### 3.2. Tissue Distribution of Coronaviruses

A total of 1490 tissues were received from the 365 animals. To reduce the number of tissues requiring testing, we first screened all tissues from the first 96 animals submitted, including 72 rodents (50 deer mice and 22 house mice), 13 mink, 5 raccoons, and 6 striped skunks. A total of 459 tissues or tissue pools (multiple tissues were collected into the same tube at necropsy) were tested for SARS-CoV-2 from these animals (Supplementary Table S1). Eleven percent of the lungs and 24% of the colon/rectum pools were positive for SARS-CoV-2 (Table 2). None of the other tissues tested were positive for SARS-CoV-2.

We focused on the lung and colon/rectum pool samples for the remainder of the study, although some additional tissues were also tested. A total of 261 animals had at least one tissue positive by the RT-PCR tests. From these, 127 alphacoronaviruses and 88 betacoronaviruses were identified either by SARS-CoV-2-specific RT-PCR or by pan-coronavirus RT-PCR testing and sequence analysis (Table 3). There were 74 tissues that were pan-coronavirus RT-PCR positive but had insufficient material for sequence identification. We identified two alphacoronaviruses from two feral domestic cats and a betacoronavirus from a deer mouse. There were three other deer mice that had the correct sized amplicon but could not be sequence-confirmed. We found two alphacoronaviruses and 13 betacoronavirus in the house mice with six other RT-PCR positives that could not be further characterized. One hundred and twenty-three alphacoronaviruses and 74 betacoronaviruses were found in mink samples with an additional 60 samples that were RT-PCR positive for coronavirus but that could not be further characterized. Finally, two raccoon and three skunk samples were similarly RT-PCR positive but could not be further characterized.

### 3.3. Phylogenetic Analysis

**Figure 1:**
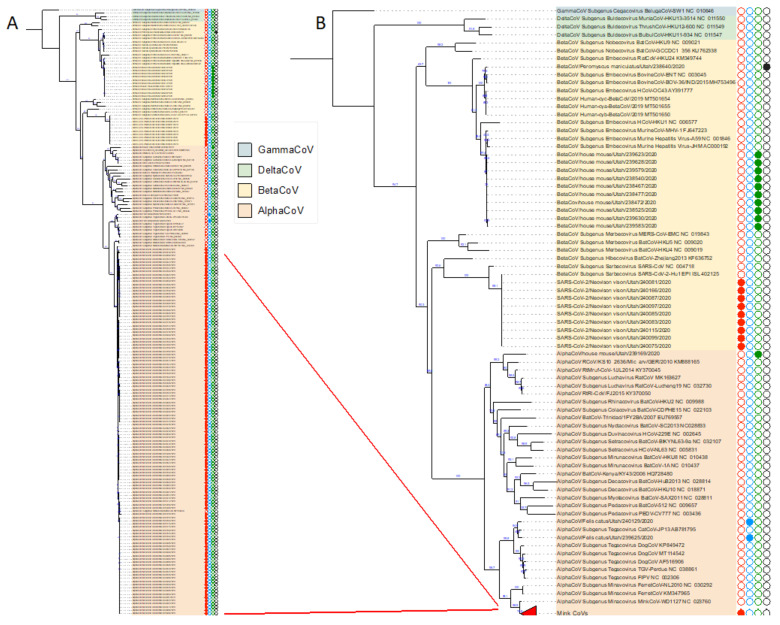
Phylogenetic relationships of the identified coronaviruses from mink and other animals from mink farms in Utah. The four genera of coronaviruses are highlighted in different colors. AlphaCoV, alphacoronavirus; BetaCoV, betacoronavirus; DeltaCoV, deltacoronaviruses, and GammaCoV, gammacoronavirus. Type species for the currently recognized subgenera are with the ICTV subgenus, strain name and the GenBank locus name. Panel A. Full phylogenetic tree (A full-size image is included in Supplementary Figure S1). Red lines designate the group of nearly identical Utah mink coronavirus strains collapsed into the colored triangle in Panel B. Circles denote animal species; red, American mink; blue, cat; green, house mouse; black, deer mouse. Filled circles denote strains characterized in this study.

We sequenced the 512 basepair region of the RNA-dependent RNA polymerase (RdRp) gene that is amplified by the broad-specificity coronavirus RT-PCR assay and compared them to previously known coronavirus sequences by BLAST and phylogenetic analyses. As expected, all the alphacoronaviruses and betacoronaviruses identified in this study fall into their respective coronavirus subgenera (Figure 1, Supplementary Figure S1). The two alphacoronaviruses identified from feral domestic cats were identified as feline coronavirus. All the mink alphacoronavirus sequences were closely related to each other and to published mink coronavirus sequences, while the alphacoronaviruses from the house mouse were related to other rodent alphacoronaviruses such as AlphaCoV/*Mydes rufocanus*/Jilan/RtMruf-CoV-1/2014 (GenBank Accession KY370045). The betacoronaviruses found in the house mice were related to murine hepatitis virus (MHV). The betacoronavirus identified in a white-footed deer mouse was an outlier and was most similar to a group of bovine and human coronaviruses, OC43 (Figure 1). Of the 74 betacoronaviruses found in mink, all were positive by the CDC N1 assay and sequencing of ten samples showed near identity to contemporary circulating strains such as SARS-CoV-2/*Felis catus*/USA/TAMU-078/2020 (GenBank Accession MW263337). 

### 3.4. Coinfections

At least 27 mink were coinfected with SARS-CoV-2 and a second coronavirus (Supplementary Table S2). An additional 16 mink may have had a co-infection with SARS-CoV-2 and a second coronavirus that we could not further characterize. Of the 27 mink for which the identity of the second coronavirus could be confirmed by sequence analysis, all were co-infected with a mink alphacoronavirus (Table 4).

## 4. Discussion

As part of a collaborative One Health-based investigation to characterize and understand the dynamics of Severe Acute Respiratory Syndrome Coronavirus-2 (SARS-CoV-2) outbreaks in people and multiple animal species on mink farms in Utah, 365 farmed or free-ranging mammals found on and around affected farms were sampled to assess SARS-CoV-2 infection status and to identify infections or co-infections by other coronaviruses. For this analysis, no attempt was made to distinguish whether mink trapped adjacent to the farms were freely living wild animals or farm escapees. While at least one mink in this study had been shown to be wild (Supplementary Table S1) [9], all of the mink trapped during this study were potentially able to interact with other wildlife. Our study used a broad-specificity RT-PCR test that can detect SARS-CoV-2 in addition to all four genera of coronaviruses and a SARS-CoV-2-specific test. A more comprehensive study is planned to separately examine the detailed distribution of SARS-CoV-2 among these animals and include sample types not tested in this study. It is interesting to note that we did not detect presence of SARS-CoV-2 in any species other than mink (Supplementary Table S2). SARS-CoV-2 infection of farmed mink was first reported in the Netherlands [15]; and as of Jul 20, 2021, 435 mink farms in 12 countries, including the United States, have been infected [9]. Zoonosis from exposure to infected farm workers is suspected to be the source of transmission to the mink [2], and in some cases zoonotic transmission from captive mink to farm workers has been demonstrated by the genetic relationship of their viruses [16,17]. The lack of SARS-CoV-2 detection in the other animal species examined for this study could mean that sampled animals were not exposed to any infected mink, that the species sampled were resistant to infection by this virus, or that infections were mild or localized and did not lead to detectable viral burden in the tissues tested.

Most mammalian coronaviruses belong to the alpha- and beta-coronavirus genera, and through this effort, we identified coronaviruses in the mammals from Utah that belonged to both groups. All 123 alphacoronaviruses identified in mink (e.g., AlphaCoV/*Neovison vison*/Utah/239637/2020) were closely related to an alphacoronavirus previously described from mink*. This alphacoronavirus, now named mink coronavirus-1 (MCoV-1), is associated with catarrhal gastroenteritis in mink and was first described by Vlasova et al. [18]. The mink coronavirus in the present study is most closely related to an AlphaCoV/mink/China/1/2016 (GenBank accession MF113046) and to AlphaCoV/mink/Minnesota/WD1133/1998 (GenBank accession HM245926). Both of these coronaviruses, in turn, are related to AlphaCoV/mink/Wisconsin/WD1127/1998 (GenBank accession NC_023760), which is the reference sequence for MCoV-1. Whether infection of the Utah mink with MCoV-1 is associated with gastrointestinal pathology is presently unknown. Both the American mink and the domestic ferret belong to the family *Mustelidae*. Both species are susceptible to SARS-CoV-2, but they have different suites of endogenous coronaviruses. Until the recent identification of SARS-CoV-2 infection in mink, all previous coronaviruses found in mink and ferrets were in the genus *Alphacoronavirus*, subgenus *Minacovirus* [8]. While published information about coronaviruses in wild mustelids is currently limited, this study indicates intense circulation of the mink coronavirus (detected in 91% of tested animals) in and around the proximity of mink farms within our study area. This observation raises the possibility of reassortment between the alpha- and betacoronaviruses and SARS-CoV-2, and the generation of novel animal coronaviruses with altered host specificity and pathogenicity in the animals studied.

A different alphacoronavirus was identified in two house mice (AlphaCoV/house mouse/Utah/239169/2020). This virus is most closely related to a coronavirus identified in voles in China (AlphaCoV/*Myodes rufocanus*/China/RtMruf-CoV-1/2014, GenBank accession KY370045). Together these viruses are in the subgenus *Luchacovirus*. The first member of the Luchacovirus group was identified in a rat (*Rattus norvegicus*) from Zhejiang, China [19]. Additional members were subsequently found in voles and rats elsewhere in China and the United Kingdom [20,21]. Tsoleridis et al. reported that an alphacoronavirus identified from rats, mice, and shrews in Europe, together with the Luchacovirus, formed a distinct rodent/shrew clade of alphacoronavirus [22]. We extend these findings to identify the first members of Luchacovirus in North America.

Alphacoronaviruses have previously been found in animals with possible associations with farms. For example, a coronavirus was identified in a moribund raccoon in the United States [23]; and in Japan, 7% of wild raccoons are sero-positive for canine alphacoronavirus [24]. While Bosco-Lauth et al. showed that skunks but not raccoons are susceptible to infection with SARS-CoV-2, prior to this study, natural coronavirus infection had not been reported in skunks [25]. In our study, we detected the presence of coronavirus RNA in two raccoons and three skunks but were unable to characterize the viruses further. Two examples of farm cats infected with the feline coronavirus were identified through our study, however, further work is necessary to characterize these viruses as feline enteric coronavirus (FECV) or feline infectious peritonitis virus (FIPV). During the SARS-CoV-2 pandemic, domestic cats and dogs, lions (*Panthera leo*), pumas (*Puma concolor*), snow leopards (*Panthera uncia*) and tigers (*Panthera tigris*) in zoological parks have been infected with the SARS-CoV-2 [9,26,27,28]. The extent of the SARS-CoV-2 host range in companion and other animals is incompletely known.

Eleven of the betacoronaviruses identified from house mice (e.g., BetaCoV/house mouse/Utah/2384726/2020) were closely related to each other and to murine coronavirus-1 (MCoV-1), which was initially called Murine Hepatitis Virus (MHV). MHV was thought to be the cause of fatal hepatitis in mice, but the pathology was found to be variable depending on the genetics of the mice and viral lineage [29]. Recently, MHV has been used as a murine model for Severe Acute Respiratory Syndrome Coronavirus (SARS) in A/J mice [30]. MHV has been previously identified in wild mice in the United Kingdom and the United States, but little is known about its species distribution or patterns of transmission [31,32].

A final virus that we have identified is a betacoronavirus from a deer mouse. BetaCoV/*Peromyscus maniculatus*/Utah/238640/2020 was most closely related to bovine coronaviruses, and both cluster with human respiratory coronavirus OC43. This group of coronaviruses was suggested to be an example of “promiscuous” coronaviruses by Drexler et al. [33], because OC43-related viruses have been found in antelope, camels, cows, deer, dogs, giraffes, horses, and people. Identification of an OC43 subgroup virus in deer mice provides further evidence of the wide host range of this group of betacoronaviruses. A recent survey of Laotian wildlife found rodent coronaviruses in two distinct genetic clusters, one of which is related to OC43 [34]. Monchatre-Leroy et al. found one rodent and five rabbit coronaviruses in France that belong to this group [35]. While both MHV and OC43 coronaviruses are in the subgenus *Embecovirus*, a study showed that deer mice were refractory to experimental challenge with MHV. Further experimental infections with the viruses identified in the current study might help address the question of whether the subgenus *Embecovirus* requires further differentiation based on host range and species susceptibility [36]. 

We caution that taxonomic relationships of the coronaviruses detected in Utah mammals through this study were determined using only a portion of the RNA-dependent RNA-polymerase (RdRp) gene. While this gene has been widely used in the literature for taxonomic assignment of coronavirus taxonomy, analyzing a longer region of the RdRp gene, together with additional protein coding regions of the genome would help determine final taxonomic assignments. Moreover, because coronaviruses are known to recombine, full genome analyses would be useful to fully appreciate the potential diversity of the coronaviruses identified through this study. Finally, only limited information has been published on coronaviruses from wild mammals of the United States. Thus, additional animals and locations would need to be sampled to fully assess coronavirus diversity in these species. 

As exemplified by the emergence of SARS-CoV-2, information on coronaviruses harbored by animals in the context of the ecological roles of the sampled species can inform risk assessments for such viruses to become a threat to wildlife, production animals, domestic animals, or public health. Our findings indicate an unexpectedly high prevalence of coronavirus among the domestic and wild animals tested on mink farms and raise the possibility that these operations could be potential hot spots for future trans-species viral spillover and the emergence of new pandemic coronaviruses. Further research on emerging coronaviruses including communities and populations associated with farm environments where susceptible animals are raised is a central component of a One Health approach and is crucial to preventing the introduction and spread of SARS-CoV-2 to people, mink, and other susceptible animals [37].

## Figures and Tables

**Table 1:** Coronavirus distribution among species tested. The species are listed by their common names; Total, the total number of animals of each species tested; Negative, number of each species with no coronavirus detected among the tissues tested; Positive, number of animals positive for coronavirus in at least one tissue; % Pos, percentage of coronavirus positives in each species.

Species	Total	Negative	Positive	% Pos
Cat	2	0	2	100%
Mink	252	23	229	91%
Mouse, Deer	47	43	4	9%
Mouse, House	51	30	21	41%
Raccoon	6	4	2	33%
Skunk, Striped	7	4	3	43%
Total	365	104	261	72%

**Table 2:** Detailed tissue panel tested for SARS-CoV-2. The distribution of SARS-CoV-2 RNA detection in the first 96 animals is listed. Tissue, tissue or tissue pools received; Total, total number tested in each category; Negative, number of N1 RT-PCR negatives; Positives, number of N1 RT-PCR positives; % Pos, percentage of tissues positive for coronavirus.

Tissue	Total	Negative	Positive	% Pos
Colon/Rectum	89	68	21	24%
Heart/Kidney	90	90	0	-
Liver	48	48	0	-
Liver/Spleen	42	42	0	-
Lung	96	85	11	11%
Small Intestine	89	89	0	-
Trachea	5	5	0	-
Total	459	427	32	7%

**Table 3:** Summary of coronaviruses identified. The distribution of coronaviruses detected and characterized according to their host is listed. Species, common name of animal species tested; AlphaCoV, number of alphacoronaviruses identified; BetaCoV, number of betacoronaviruses identified; Sequenced, number of viruses identified by sequencing, Unchar, number of coronavirus-positive samples not further characterized.

Species	AlphaCoV	BetaCoV	Sequenced	Unk
Cat	2	0	2	0
Mink	123	74	131	60
Mouse, Deer	0	1	1	3
Mouse, House	2	13	15	6
Raccoon	0	0	0	2
Skunk, Striped	0	0	0	3
Total	127	88	149	74

**Table 4:** SARS-CoV-2 coinfections identified in Utah mammals. The individual animals that are both SARS-CoV-2 positive and infected with a second coronavirus are listed. Animal ID, Unique animal identification number; Common name, common name of animal; Scientific name, scientific name of animal; Sex, F, female, M, male. Unk, unknown; Age, A adult, J juvenile, Unk, unknown; SARS-CoV-2, Neg-N1 RT-PCR negative, Pos-N1 RT-PCR positive, Second strain, genus and common name of the coronavirus, Pan-CoV RT-PCR Equivocal, sample is PCR positive but not further characterized.

Animal ID	Common Name	Scientific Name	Sex	Age	SARS-CoV-2	Second Strain
46844-098	Mink	*Neovison vison*	M	J	Pos	AlphaCoV (MinkCoV)
46844-100	Mink	*Neovison vison*	M	A	Pos	AlphaCoV (MinkCoV)
46844-105	Mink	*Neovison vison*	M	J	Pos	AlphaCoV (MinkCoV)
46844-118	Mink	*Neovison vison*	M	A	Pos	AlphaCoV (MinkCoV)
46844-124	Mink	*Neovison vison*	M	UNK	Pos	AlphaCoV (MinkCoV)
46844-125	Mink	*Neovison vison*	F	J	Pos	AlphaCoV (MinkCoV)
46844-151	Mink	*Neovison vison*	M	A	Pos	AlphaCoV (MinkCoV)
46844-154	Mink	*Neovison vison*	M	A	Pos	AlphaCoV (MinkCoV)
46844-155	Mink	*Neovison vison*	M	A	Pos	AlphaCoV (MinkCoV)
46844-164	Mink	*Neovison vison*	F	J	Pos	AlphaCoV (MinkCoV)
46844-165	Mink	*Neovison vison*	M	J	Pos	AlphaCoV (MinkCoV)
46844-168	Mink	*Neovison vison*	M	J	Pos	AlphaCoV (MinkCoV)
46844-169	Mink	*Neovison vison*	M	J	Pos	AlphaCoV (MinkCoV)
46844-177	Mink	*Neovison vison*	F	J	Pos	AlphaCoV (MinkCoV)
46844-202	Mink	*Neovison vison*	M	A	Pos	AlphaCoV (MinkCoV)
46844-208	Mink	*Neovison vison*	M	A	Pos	AlphaCoV (MinkCoV)
46844-212	Mink	*Neovison vison*	M	A	Pos	AlphaCoV (MinkCoV)
46844-214	Mink	*Neovison vison*	M	J	Pos	AlphaCoV (MinkCoV)
46844-217	Mink	*Neovison vison*	F	A	Pos	AlphaCoV (MinkCoV)
46844-220	Mink	*Neovison vison*	M	A	Pos	AlphaCoV (MinkCoV)
46844-221	Mink	*Neovison vison*	F	UNK	Pos	AlphaCoV (MinkCoV)
46844-236	Mink	*Neovison vison*	UNK	UNK	Pos	AlphaCoV (MinkCoV)
46844-243	Mink	*Neovison vison*	M	J	Pos	AlphaCoV (MinkCoV)
46844-248	Mink	*Neovison vison*	M	A	Pos	AlphaCoV (MinkCoV)
46844-257	Mink	*Neovison vison*	UNK	UNK	Pos	AlphaCoV (MinkCoV)
46844-291	Mink	*Neovison vison*	F	A	Pos	AlphaCoV (MinkCoV)
46844-297	Mink	*Neovison vison*	F	A	Pos	AlphaCoV (MinkCoV)
46844-103	Mink	*Neovison vison*	F	J	Pos	Pan-CoV Equivocal
46844-111	Mink	*Neovison vison*	F	A	Pos	Pan-CoV Equivocal
46844-115	Mink	*Neovison vison*	F	A	Pos	Pan-CoV Equivocal
46844-188	Mink	*Neovison vison*	F	J	Pos	Pan-CoV Equivocal
46844-203	Mink	*Neovison vison*	M	A	Pos	Pan-CoV Equivocal
46844-210	Mink	*Neovison vison*	F	A	Pos	Pan-CoV Equivocal
46844-226	Mink	*Neovison vison*	F	UNK	Pos	Pan-CoV Equivocal
46844-249	Mink	*Neovison vison*	F	A	Pos	Pan-CoV Equivocal
46844-259	Mink	*Neovison vison*	UNK	UNK	Pos	Pan-CoV Equivocal
46844-260	Mink	*Neovison vison*	UNK	UNK	Pos	Pan-CoV Equivocal
46844-261	Mink	*Neovison vison*	UNK	UNK	Pos	Pan-CoV Equivocal
46844-262	Mink	*Neovison vison*	F	UNK	Pos	Pan-CoV Equivocal
46844-267	Mink	*Neovison vison*	F	A	Pos	Pan-CoV Equivocal
46844-281	Mink	*Neovison vison*	F	A	Pos	Pan-CoV Equivocal
46844-282	Mink	*Neovison vison*	F	A	Pos	Pan-CoV Equivocal
46844-283	Mink	*Neovison vison*	M	A	Pos	Pan-CoV Equivocal

* As a side note, there is no standard nomenclature for describing strains of coronavirus. In this paper we will use the following format so as to make references to strains more consistent: Genus/host species/location/laboratory strain ID/year. For example: “Mink coronavirus strain WD1133 isolated from mink in Minnesota in 1998 will be AlphaCoV/mink/Minnesota/WD1133/1998.

## Data Availability

The data generated by this study are fully disclosed in the text and in the Supplementary Materials. The data also are available at https://doi.org/10.5066/P9X5VR9S.

## Supplementary Materials

The following are available online at https://www.mdpi.com/article/10.3390/v13102016/s1. Phylogenetic relationships of the identified coronaviruses from mink farms in Utah. Table S1: List of animals and tissues sampled and RT-PCR test results. Table S2: Summary of coronavirus test results.
