# Supplementary material for: An Opportunistic Survey Reveals an Unexpected Coronavirus Diversity Hotspot in North America
Source: Viruses. 2021 Oct 7;13(10):2016. doi: 10.3390/v13102016 (PMC8539472; doi:10.3390/v13102016)
Supplement: Supplementary file 1 [file viruses-13-02016-s001.zip › 211004 Supplementary Figure Legends.pdf]

viruses

MDPI

Supplementary File Legends

Supplementary Figure S1. Phylogenetic relationships of the identified coronaviruses from mink farms in Utah. The four genera of coronaviruses are highlighted in different colors. AlphaCoV, alphacoronavirus; BetaCoV, betacoronavirus; DeltaCoV, deltacoronaviruses; and GammaCoV, gammacoronavirus. Type species for the currently recognized subgenera are annotated according to the nomenclature scheme used in this manuscript. Filled circles denote the animal species from which strains were characterized in this study; red, American mink; blue, cat; green, house mouse; black, deer mouse.

Supplementary Table S1. List of animals and tissues sampled and RT-PCR test results. Animal ID, unique identifier for each animal; Specimen ID, unique identifier for each tissue; Common name, common name of the animal species; Scientific name, scientific name of the animal species; Sex, F-female, M-male, UNK-unknown; Age, J-juvenile, A-adult, UNK-unknown; Tissue, organ or organ pools tested; Tissue study, X denotes the animals and tissues used in the tissue distribution sub-study; N1 PCR, Ct values from the CDC N1 assay; Pan-CoV PCR, Neg, negative, Pos, positive, Equiv, equivocal; * wild mink.

Supplementary Table S2. Summary of coronavirus test results. Animal ID, unique identifier for each animal; Common name, common name of the animal species; Scientific name, scientific name of the animal species; Sex, F-female, M-male, UNK-unknown; Age, J-juvenile, A-adult, UNK-unknown; CoV, Neg-negative, Pos-positive on either one or both pan-coronavirus RT-PCR tests; SARS-CoV-2, Pos-animals positive in the CDC N1 test, Neg-animals negative in the CDC N1 test; AlphaCoV, the tissues positive for alphacoronavirus for each animal is listed; BetaCoV, the tissues positive for betacoronavirus for each animal is listed; C-colon, C/R-colon/rectum pool, H-heart, L-lung, L/S-live/spleen pool, S int-small intestine; Co-infections, Y-yes; PCR only, Y-yes; Virus identified by sequencing, brief name of virus identified.
