# Supplementary figures and images for: An Opportunistic Survey Reveals an Unexpected Coronavirus Diversity Hotspot in North America
Source: Viruses. 2021 Oct 7;13(10):2016. doi: 10.3390/v13102016 (PMC8539472; doi:10.3390/v13102016)

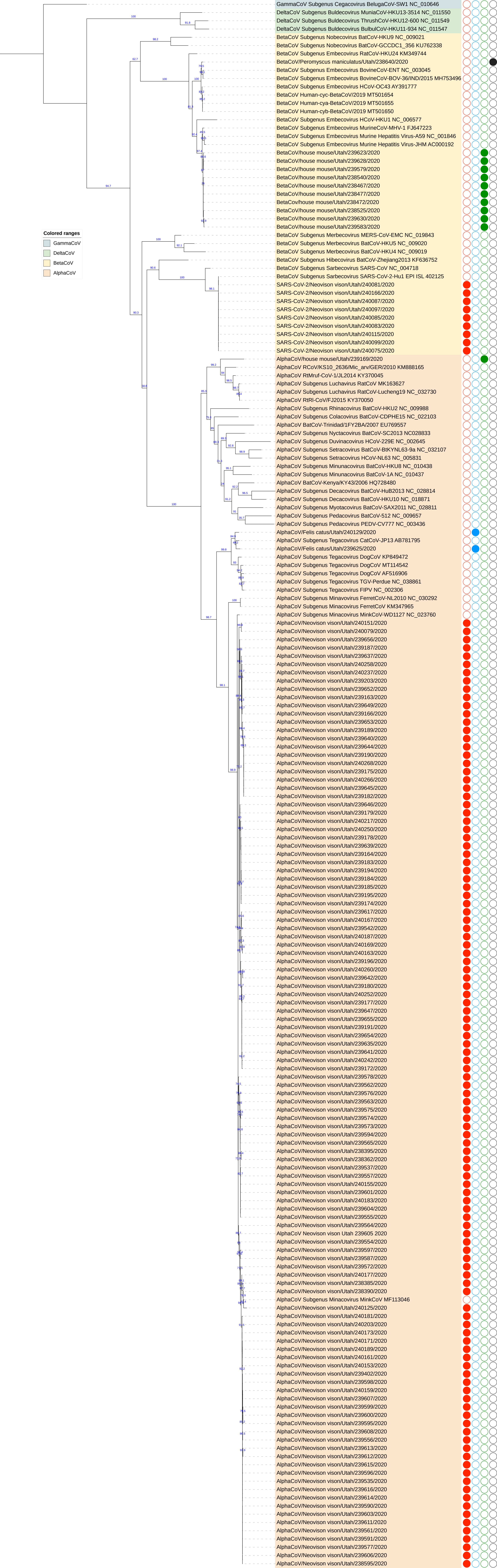

Supplement: Supplementary file 1 [file viruses-13-02016-s001.zip › 211004 Supplementary Figure S1.pdf]
